# Water Calorimetry: The Heat Defect

**DOI:** 10.6028/jres.102.006

**Published:** 1997

**Authors:** Norman V. Klassen, Carl K. Ross

**Affiliations:** Ionizing Radiation Standards, Institute for National Measurement Standards, National Research Council Canada, Ottawa K1A 0R6 Canada

**Keywords:** absorbed dose to water, dosimetry, ionizing radiation, water calorimetry

## Abstract

Domen developed a sealed water calorimeter at NIST to measure absorbed dose to water from ionizing radiation. This calorimeter exhibited anomalous behavior using water saturated with gas mixtures of H_2_ and O_2_. Using computer simulations of the radiolysis of water, we show that the observed behavior can be explained if, in the gas mixtures, the amount-of-substance of H_2_ and of O_2_ differed significantly from 50 %. We also report the results of simulations for other dilute aqueous solutions that are used for water calorimetry—pure water, air-saturated water, and H_2_-saturated water. The production of H_2_O_2_ was measured for these aqueous solutions and compared to simulations. The results indicate that water saturated with a gas mixture containing an amount-of-substance of H_2_ of 50 % and of O_2_ of 50 % is suitable for water calorimetry if the water is stirred and is in contact with a gas space of similar volume. H_2_-saturated water does not require a gas space but O_2_ contamination must be guarded against. The lack of a scavenger for OH radicals in “pure” water means that, depending on the water purity, some “pure” water might require a large priming dose to remove reactive impurities. The experimental and theoretical problems associated with air-saturated water and O_2_-saturated water in water calorimeters are discussed.

## 1. Introduction

Pure water and a number of dilute aqueous solutions have been used as the absorbing media in water calorimeters designed to measure absorbed dose to water from ionizing radiation. Recently, Domen [1] found large variations in the temperature rise per unit dose using a sealed water calorimeter containing a tiny gas space and motionless water that was saturated with what was intended to have been equal flow rates of H_2_ and O_2_. Just such a solution has been found to be an excellent absorbing medium in a stirred water calorimeter with a large gas space [2–5]. Therefore, we investigated and found the reason for this apparent discrepancy.

Water calorimetry [1, 4–7] measures the absorbed dose to water from ionizing radiation using the temperature rise produced in water and the equations

$$D_{w}=\left(c_{w}\Delta T\right)/\left(1-k_{\mathrm{HD}}\right)$$ (1)

$$\text{and}\;k_{\mathrm{HD}}=\left(E_{a}-E_{h}\right)/E_{a},$$ (2)

where *D*_w_ is the absorbed dose to the water, *c*_w_ is the specific heat capacity of the water, **Δ***T* is the temperature rise, *k*_HD_ is a correction called the heat defect, *E*_a_ is the energy absorbed by the water, and *E*_h_ is the energy which appears as heat. The heat defect corrects for the radiation-induced chemical changes in the water, which cause the measured temperature rise to be greater or smaller than the value corresponding to complete conversion of *E*_a_ into heat. Using the radiation yields of primary products and the ensuing chemical reactions, the chemical changes in irradiated solutions can be simulated using computer models and the heat defects can be calculated.

Previously, the calculated heat defects were verified by comparing the relative temperature rises in a variety of solutions [2, 5]. Another way to verify the calculated heat defects is to measure the chemical products of radiolysis and compare them to the model calculations.

In this study, the production of H_2_O_2_ was measured and compared to model predictions for a variety of solutions. Also reported are investigations into the serious problems associated with the use of air- and O_2_-saturated water and into the effect of traces of O_2_ in pure water and in H_2_-saturated water.

## 2. Experimental

Water was purified by passage through a charcoal filter, a Millipore RO10 (reverse osmosis) unit^1^, and a Millipore Milli-Q UV unit, in that order. The charcoal filter removed suspended solids. The Millipore RO10 removed about 95 % of the dissolved impurities. The Milli-Q UV unit contains activated charcoal, ion exchange resins and an organic scavenger and, as a final treatment, the unit photolyses the water with 184 nm and 254 nm light from a low-pressure mercury vapor UV lamp to oxidize organic impurities, mainly to carbon dioxide and water, and the water exits the unit through a 0.22 μm filter. The Milli-Q UV produces water with a resistivity of 18.2 × 10^6^ Ω · cm at a rate of 1.2 L min^−1^. Water taken from the Milli-Q UV was stored in well-cleaned quartz vessels. Water could also exit the Milli-Q UV through another port and go directly to an Anatel A-10 TOC (total organic carbon) monitor [8] to be analyzed for organic impurities. Typically, the A-10 gave a reading of “3 ppb,” which means a mass concentration of 3 μg L^−1^ of organic carbon, which is equivalent to an amount-of-substance concentration of 2.5 × 10^−7^ mol L^−1^ of organic carbon in the water as it left the Milli-Q UV unit. This would be equivalent to an amount-of-substance concentration of 1.25 × 10^−7^ mol L^−1^ of organic impurity if, for example, the organic impurity were a compound containing 2 carbon atoms. The A-10 monitor gives spurious readings for total organic carbon if the water has previously picked up carbon dioxide from the air. Hence, it could not be used to determine the increase in the total organic carbon in the water after storage or irradiation.

Water was saturated with gases by bubbling the gas, or gas mixture, through the water for 30 min to 60 min at a rate of 200 cm^3^ min^−1^. Gas flow rates were measured using Matheson model 8141 mass flowmeters which were calibrated using an MKS Califlow system. The uncertainty^2^ in the flow rates was less than 1 %. The solubilities of H_2_ and O_2_ in water were taken from the literature [9, 10]. Only high purity gases were used. These gases, including the manufacturers’ stated impurities and their concentrations expressed as a mole fraction of the gas, were: hydrogen (Air Products, Ultrapure Carrier Grade < 0.1 × 10^−6^ of O_2_ and < 1.0 × 10^−6^ of total hydrocarbons), oxygen (Air Products, Ultrapure Carrier Grade < 0.5 × 10^−6^ of total hydrocarbons), nitrogen (Air Products, Ultrapure Carrier Grade < 1.0 × 10^−6^ of O_2_, < 0.5 × 10^−6^ of total hydrocarbons) and argon (Canadian Liquid Air, Super Purified grade, < 0.1 × 10^−6^ of O_2_, < 0.5 × 10^−6^ of total hydrocarbons).

Our calorimeter vessels are made of thin (0.18 mm to 0.67 mm) Pyrex and contain 100 mL of stirred water in a cylindrical volume about 5 cm in diameter and 5 cm high. The gas space, above the water, is about 90 cm^3^. The water temperature was measured using two independent thermistor probes. The calorimeter vessel is entirely surrounded by a shroud regulated to about ± 0.5 mK. Detailed descriptions of the calorimeter have been published [4,11].

Calorimetry measurements, using ^60^Co beams, consisted of a set of 10 irradiations of 210 s duration each at a dose rate of about 7.2 cGy s^−1^ resulting in a temperature rise of about 3.6 mK for each irradiation. Irradiations using the linear accelerator were carried out with the x rays (for convenience, called 20 MV photons) which result when a beam of 20 MeV electrons is directed into a fully-stopping aluminum block. Using the linear accelerator, a series of irradiations of 48 s duration each at 42 cGy s^−1^ were done, resulting in a temperature rise of about 4.8 mK for each irradiation. Dosimetry, for irradiations done in the calorimeter, was based on water calorimetry. For other irradiations, dosimetry was done by Fricke dosimetry using a value of 350.5 × 10^−6^ m^2^ J^−1^ for *εG*(Fe^3+^)^3^ and applying the appropriate corrections for the temperatures of the irradiations and the optical density measurements [13].

H_2_O_2_ was measured by the $I_{3}^{-}$ method in which H_2_O_2_ converts KI into $I_{3}^{-}$ in the presence of a catalyst. The concentration of $I_{3}^{-}$ was measured spectrophotometrically. The uncertainty in the analysis was about ± 0.03 μmol L^−1^ of H_2_O_2_ [14].

The measurement of O_2_ in water, or in gas in equilibrium with water, was done using an EIT (Enterra Instrumentation Technologies) model 5121 Dissolved Oxygen Monitor. The EIT monitor registers the concentration of O_2_ in water in “ppb” and has a detection limit of “0.1 ppb.” The value in units of “ppb” corresponds to the mass fraction of O_2_ in water in μg (kg of solution)^−1^ but the readings were converted into the amount-of-substance concentration, mol L^−1^, for ease of use. The EIT monitor was used between the detection limit (3 × 10^−9^ mol L^−1^ of O_2_) and 2.8 × 10^−4^ mol L^−1^ of O_2_ (which corresponds to air-saturated water). After 3 days of use, the readings were stable and always returned to 0.0 (± 6) × 10^−9^ mol L^−1^, of O_2_ when the probe was left overnight in saturated sodium sulfite solution. When pure H_2_ or N_2_ was bubbled through water in contact with the probe, a reading of 4 × 10^−9^ mol L^−1^ of O_2_ was attainable as compared to ≤ 1 × 10^−10^ mol L^−1^ which corresponds to the stated gas purities. The monitor can also be used to measure the oxygen content of a gas, a feature which was used to measure the concentration of O_2_ in a remote water sample (e.g., the calorimeter vessel) which was not in contact with the probe. This was done by bubbling a gas through the remote water sample to bring the oxygen content of the water and the gas into equilibrium. The gas was then flowed over the membrane of the probe. The probe gives the same reading when it is exposed to either gas or water whose concentrations of oxygen are in equilibrium. In this way, the concentration of O_2_ in the water in the calorimeter was monitored. If the monitor readings started at a high level, e.g., 2.8 × 10^−4^ mol L^−1^ of O_2_, the response to a low level, e.g., 3 × 10^−8^ mol L^−1^ of O_2_, was very slow. In order to get a fast response (less than 1 min) to low levels of O_2_, the probe was continuously purged with pure H_2_ or N_2_ to keep the monitor reading low in between the periods when it was switched over to the gas whose O_2_ content was to be measured.

## 3. Results and Discussion

### 3.1 The Model

A model reaction scheme was used to simulate the radiolysis of water and aqueous solutions. The model, as shown in Table 1, contains 50 reactions and their rate constants. The *G*-values of the primary products are shown in Table 2 for 21 °C and for 25 °C. The model is revised from time to time and we are now using version III. Comparisons between version III and version II [5] are made in this report. Equations which account for the transfer of gases between the gas phase and the stirred water, at the rates at which it occurs in our calorimeter, have been reported [5]. These were used in the simulations, when appropriate, but are not included in Table 1. Although our irradiations were carried out at 21 °C, the rate constants at 25 °C, as proposed by Elliot [15], were used because a self-consistent set could be more closely approximated for 25 °C than for 21 °C. For the aqueous solutions used in this study the rate constants at 25 °C are quite acceptable. However, unless indicated otherwise, the *G*-values for 21 °C, not those for 25 °C, were used for the simulations reported here because the values for 21 °C are believed to be closer to the values for our solutions. It is not precluded to use the *G*-values for 21 °C and the rate constants for 25 °C because there is no simple link between the temperature dependence of the two sets of parameters.

The primary radiolytic species are produced close to one another in “spurs,” and some react with each other very quickly by spur reactions that follow nonhomogeneous kinetics [16, 17]. The dilute solutes used in this work do not interfere with the spur reactions. At times longer than 10^−7^ s after spur formation, the reactions follow homogeneous kinetics, i.e., the reactive species behave as if they were homogeneously distributed in the solution [15]. The radiolysis of solutions containing low concentrations of solutes, at dose rates typical of water calorimetry, can be simulated using the *G*-values at 10^−7^ s. These *G*-values, for 21 °C, the temperature of our calorimeter, were calculated from Elliot’s report [15] and are shown in Table 2. In addition to Elliot’s values, we use *G*(OH^−^) = 0.43 and $G\left(H^{+}\right)=G\left(e_{\mathrm{aq}}^{-}\right)+G\left({\mathrm{OH}}^{-}\right)=3.06$ for low LET radiation [18].

The enthalpies of the overall chemical changes in irradiated solutions can be exothermic, resulting in a temperature rise which is greater than that corresponding to complete conversion of the absorbed dose into heat, or endothermic, resulting in a temperature rise which corresponds to less than complete conversion. If the overall chemical change is exothermic, the heat defect is negative and if the chemical change is endothermic, the heat defect is positive. A solution, frequently referred to in this study, is water saturated with a gas mixture made up of H_2_ and O_2_ at equal flow rates, i.e., a gas mixture made up of an amount-of-substance fraction of H_2_ = 50 % and an amount-of-substance fraction of O_2_ = 50 %. We designate this as “H_2_/O_2_ water.” The radiolysis of H_2_/O_2_ water, the way it is used in our stirred water calorimeter, is 2.4 % exothermic, i.e., it has a heat defect of − 0.024. Some systems, such as pure water and H_2_-saturated water, reach a steady state after a sufficient accumulated dose. At a steady state, further absorbed dose does not change the chemical composition of the system. Therefore, once a steady state has been achieved, the differential heat defect is zero.

The calculated heat defects were tested in the past by comparing the relative temperature rises in 8 different solutions [5]. For irradiations with 20 MV photons, the relative temperature rises in the four solutions which contained a scavenger for OH radicals differed by ≤ 0.3 % with calculation if the calculated exothermicity of 2.4 % was used for H_2_/O_2_. This justifies the uncertainty we set on the value of the heat defect for H_2_/O_2_ water, which we take to be (2.4 ± 0.5) %. Another technique we have used successfully to compare calculated heat defects to experiment is to continue the calorimetry measurements to doses sufficient to convert reactive impurities into less reactive compounds, such as water and carbon dioxide, with the result that the chemistry approaches the model calculations more closely at higher doses [2].

The most direct way to determine the enthalpy changes would be to measure the chemical changes, including any vapor/liquid transformations, in the irradiated solution. This is impractical. In water calorimetry measurements, using the solutions described in this report, ≤ 1 in 10^7^ water molecules is destroyed or produced, a change not easily measured. The changes in the concentrations of H_2_ and O_2_ are of the order of 10^−6^ mol L^−1^ and are difficult to measure accurately because the separation of H_2_ and O_2_ from the solution requires vacuum techniques which are incompatible with most water calorimeters, and because large amounts of irradiated solution and larger doses than used in water calorimetry would be needed to get sufficient accuracy. H_2_O_2_ is the only product that is easy to measure without altering drastically the water calorimeter setup or irradiation protocol. Unfortunately, a knowledge of the change in H_2_O_2_ alone is insufficient for a calculation of the heat defect because it is also necessary to know how much H_2_, O_2_, and H_2_O were produced or destroyed. Nevertheless, the measurement of H_2_O_2_ in irradiated solutions is an excellent way to test model simulations and numerous examples are reported here.

If the measured value of *G*(H_2_O_2_) does not agree with the value calculated by simulation, the model can be altered, for example by changing *G*-values, in order to achieve agreement. Although such changes are slightly arbitrary, this procedure does permit an estimate to be made of the uncertainty in the calculated value of the heat defect. In order to measure *G*(H_2_O_2_) in an irradiated solution for the purpose of calculating the heat defect, both the absorbed dose and the measured concentration of H_2_O_2_ are required. The fact that the heat defect is, itself, a correction in the determination of the absorbed dose is not a problem because the heat defect is a small correction and only a few iterations are required to arrive at final values of *G*(H_2_O_2_), the heat defect, and the absorbed dose.

It is not possible to assign a reliable uncertainty to many of the rate constants or *G*-values in the model. Also, a few of the reactions in the model might involve intermediates whose existence has not yet been proven. However, the less-than-complete information about some of these reactions has not been a significant problem because most water calorimetry is based either on pure water or H_2_-saturated water, for which all models predict a heat defect of zero, or on solutions such as H_2_/O_2_ water, for which the computer simulation must provide the heat defect but for which (a) only a few reactions are important and (b) the reactive species are scavenged so that the yield of final products is not very sensitive to the values assigned to the rate constants. Even when we simulate aqueous systems for which only a few reactions in the model are significant, we usually use the whole model because (1) it is a more consistent approach, (2) it is safer in case an unsuspected reaction turns out to be important, and (3) it makes only modest demands on modern computers.

Computer simulations using model III differed insignificantly from simulations using model II except for air- or O_2_-saturated water, which are solutions we do not recommend for water calorimetry (see below). This report provides further tests of the computer simulations for H_2_/O_2_ water, for H_2_-saturated water, and for pure water. Computer simulations have now been proven to be reliable for a number of solutions and, for these solutions, simulations are the most practical way to investigate the effect of dose rate, accumulated dose, and solute concentrations on water calorimetry measurements.

### 3.2 H_2_/O_2_-Saturated Water with a Gas Space

H_2_/O_2_ water is produced by saturating water with a mixture of H_2_ and O_2_ at equal flow rates. Water calorimetry using H_2_/O_2_ water in our stirred water calorimeter has proven to be reproducible and reliable, and it constitutes our “standard” solution to which all other solutions are compared. H_2_/O_2_ water has the advantages that (a) it contains O_2_ so that trace contamination by air is no problem; and (b) it is rather insensitive to water quality because the reactive primary species are efficiently scavenged by the 7.0 × 10^−4^ mol L^−1^ of O_2_ and the 4.2 × 10^−4^ mol L^−1^ of H_2_ by three reactions:

$$e_{\mathrm{aq}}^{-}+O_{2}\to O_{2}^{-}$$ (3)

$$\mathrm{OH}+H_{2}\to H+H_{2}O$$ (4)

$$H+O_{2}\to {\mathrm{HO}}_{2}.$$ (5)

Many of the other reactions in the model participate in equilibria, which lead to no net chemical change. Significant reactions, which do produce a net change are

$${\mathrm{HO}}_{2}+{\mathrm{HO}}_{2}\to H_{2}O_{2}+O_{2}$$ (6)

and

$${\mathrm{HO}}_{2}+O_{2}^{-}{+H}_{2}O\to H_{2}O_{2}+O_{2}+{\mathrm{OH}}^{-}.$$ (7)

**Fig. 1:**
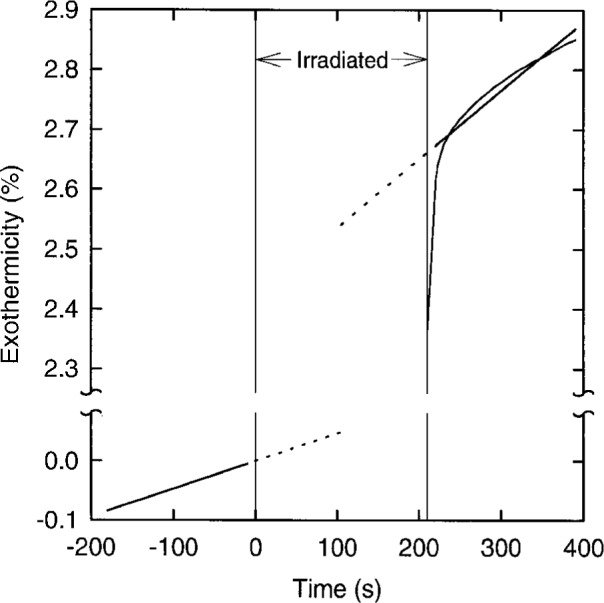
Computer simulation of the temperature rise, presented as percent exothermicity, for H_2_/O_2_ water at a dose rate of 4.1 Gy min^−1^ in the stirred water calorimeter. The simulation presented is for a single irradiation period mid-way through a set of 10 irradiation periods under the actual operating conditions of the calorimeter. Dose was delivered from 0 s to 210 s. The straight line before 0 s is the linear regression of the temperature rise between −180 s and −10 s and the percent exothermicity is set to zero at 0 s. The temperature rise before 0 s is due solely to the previous irradiations, i.e., for the first irradiation the exothermicity would be 0.0 % at all times up to the start of the irradiation. The curved line after 210 s represents the calculated percent exothermicity and the straight line between 220 s and 390 s is the linear regression of the curved line between 220 s and 390 s. The dashed lines are the extrapolations to mid-irradiation of the linear regressions.

The temperature profile during a calorimetric measurement is extrapolated to mid-irradiation to get the temperature rise. Our procedure for water calorimetry with ^60^Co beams was a set of 10 irradiation periods of 210 s duration each with pauses of 8 min between irradiations. The temperature rise is taken as the difference, at mid-irradiation, between the extrapolations of the temperature profile from 10 s to 180 s before the irradiation and 10 s to 180 s after the end of the irradiation. Exothermic changes, due to chemical reactions and to gas transfer, continue to take place to a small extent after the end of the irradiation. These changes affect the extrapolation to mid-irradiation and the value calculated for the heat defect. Most of the post-irradiation increase in exothermicity takes place during the first 10 s after the irradiation and is due mostly to chemical reactions. However, the increase continues at a slower pace after 10 s due to continued reaction and the transfer of H_2_ and O_2_ from the gas phase to the water because of the destruction of H_2_ and O_2_ in the solution caused by radiolysis. Also, a much smaller increase in temperature occurs before, during, and after an irradiation, due to transfer of H_2_ and O_2_ caused by previous irradiations of the set, an effect which builds up during the set of irradiations because, in our calorimeter, the transfer of H_2_ and O_2_ between the gas and liquid has half lives of 6 and 12 minutes for H_2_ and O_2_, respectively [5]. This much smaller rate of increase is fairly constant over the time period of a single irradiation and hence is taken care of by the extrapolation procedure without the need of a further correction. The change of exothermicity with time for a 210 s irradiation in the middle of a set was calculated from computer simulations of the chemical and physical changes; the results are shown in Fig. 1. The extrapolations are indicated by the dashed lines, which are the linear regressions of the solid lines from 10 s to 180 s, before and after the irradiation, to mid-irradiation. In Fig. 1, the exothermicity at mid-irradiation is 2.5 %, a value which is consistent with the value of (2.4 ± 0.5) % we reported for 20 MV photons [5]. Figure 1 and the above discussion make it clear that the value assigned to the heat defect for a system like H_2_/O_2_ water, which is not in a steady state, depends slightly on the duration of the irradiation and the extrapolation procedure. Therefore, the heat defect should be calculated from a simulation which duplicates the experiment. Note that the influence on the heat defect of the effects described above is less than the assigned uncertainty. As well, the change in the exothermicity of H_2_/O_2_ water in our calorimeter from the start to the finish of an irradiation set is much less than the assigned uncertainty.

In a typical irradiation period of 210 s, and at a dose rate of 4 Gy min^−1^, about 5 μmol L^−1^ of H_2_O_2_ is produced, while H_2_O, H_2_, and O_2_ are destroyed. The simulation with model III predicts 0.974 times the production of H_2_O_2_ predicted by model II, and 1.030 times the exothermicity predicted by model II. Because the heat defect is only a 2.4 % correction in calculating the dose rate, the difference between the predictions of models III and II amounts to less than a factor of 1.001 in the dose rate determined by means of water calorimetry.

**Fig. 2:**
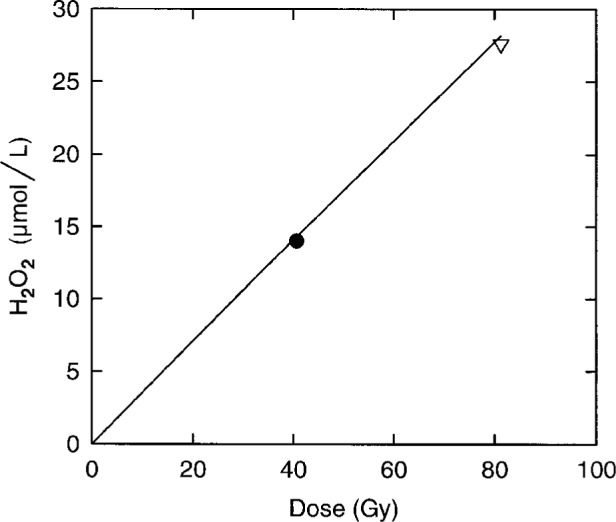
The concentration of H_2_O_2_ versus dose, for H_2_/O_2_ water in the stirred water calorimeter under normal operating conditions. The ● represents two measurements and ∇ represents a single measurement (see Table 3). The solid line represents the computer simulation using model III.

In order to compare the production of H_2_O_2_ to computer simulations, three separate fills of H_2_/O_2_ water were irradiated in the calorimeter at the usual temperature and dose rate. A dose of either 40 Gy or 80 Gy was delivered in a single irradiation period. Each solution was analyzed for H_2_O_2_. The results are shown in Table 3 and Fig. 2. The measured concentrations of H_2_O_2_ were about 2 % less than the predictions of model III and 4.5 % less than the predictions of model II. Therefore, model III is in better agreement than model II with the measured yield of H_2_O_2_. As well, replacing the *G*-values for 21 °C by the *G*-values for 25 °C in model III worsened the agreement between prediction and experiment by increasing the predicted concentration of H_2_O_2_ by 0.6 %. The simulations could be made to agree with experiment by making the *G*-values in the model 2 % smaller, which would decrease the calculated exothermicity from 2.5 % to 2.45 % and increase the dose rate measured by water calorimetry by a factor of 1.0005. Another way to force the concentration of H_2_O_2_ determined by simulation to agree with experiment would be to change rate constants in such a manner so as to reduce either, or both, the production of H_2_O_2_ from water and O_2_, or from H_2_ and O_2_. The effect on the exothermicity would depend on the method chosen. The various possibilities produce a range of exothermicities from 2.4 % to 2.5 %, all of which are consistent with the value of (2.4 ± 0.5) % we assign to the heat defect for H_2_/O_2_ water and suggest that the uncertainty we place on the value is too large. We conclude that the exothermicity of H_2_/O_2_ water under our operating conditions, and its assigned uncertainty, is now well established.

### 3.3 H_2_/O_2_-Saturated Water with No Gas Space

**Fig. 3:**
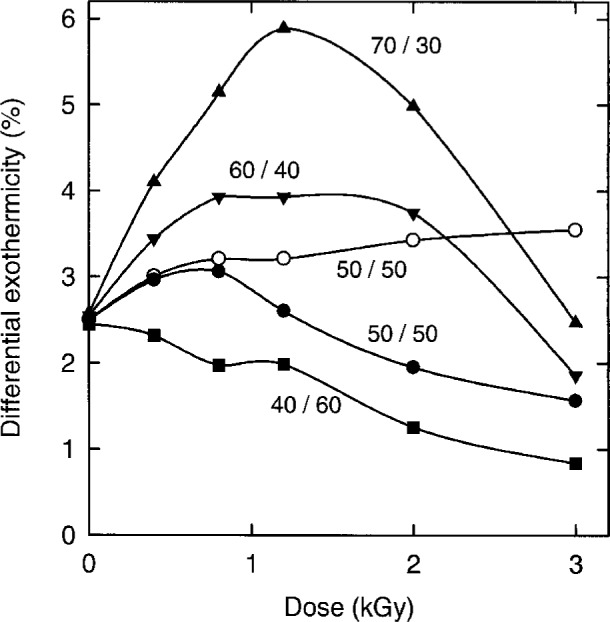
Simulations of the differential percent exothermicity for water, equilibrated with various mixtures of H_2_ and O_2_ before irradiation and irradiated in the absence of a gas space. *X*/*Y* indicates that the gas mixture used to saturate the water was made up of an amount-of-substance fraction of H_2_ = *X* % and an amount-of-substance fraction of O_2_ = *Y* %. Hence, the ratio 50/50 designates the same solution referred to in this report as H_2_/O_2_ water. Only for the simulation denoted by ○, were the concentrations of H_2_ and O_2_ in the solution forced to remain constant throughout the simulation. Otherwise, the simulations allowed the concentrations of H_2_ and O_2_ to change as dictated by the radiolysis. The differential values of the percent exothermicity were calculated for a dose of 3.1 Gy which took the accumulated dose to the dose indicated for the data point.

When H_2_/O_2_ water is irradiated, H_2_O_2_ is produced and H_2_ and O_2_ are used up. If the vessel has no gas space, H_2_ and O_2_ in the solution are removed by radiolysis but are not replaced from the gas space as they are in our stirred water calorimeter. Very large doses, given to these calorimeters, will cause large changes in the concentrations of H_2_, O_2_, and H_2_O_2_ which, in turn, will cause large changes in the exothermicity of the radiation-induced chemical changes. Figure 3 shows the results of computer simulations of the changes in the differential exothermicity, between 0 Gy and 3 kGy, as calculated for the last 3.1 Gy of the total dose. A dose rate of 1.85 Gy min^−1^, uniform across the vessel, was used for the simulations. No attempt was made to study the effects of a nonuniform dose rate across the vessel or the presence of a small gas bubble in the vessel. These effects are expected to be small and would vary from setup to setup. The predictions are shown for several H_2_/O_2_ ratios. The ratio *X*/*Y* is used here to indicate that the gas mixture used to saturate the water was made up of an amount-of-substance fraction of H_2_ = *X* % and an amount-of-substance fraction of O_2_ = *Y* %, hence, the ratio 50/50 designates the same solution referred to in this report as H_2_/O_2_ water. Other ratios in Fig. 3 were chosen to mimic some of the curves in a recent publication by Domen [1] to show that his curves arose from the use of H_2_/O_2_ ratios greater than 50/50. For example, the 70/30 curve in Fig. 3 is similar to curve 3 and curve 1 in Fig. 30 of Domen’s report and the 60/40 curve is similar to Domen’s curve 4. Doses in the kGy range drastically change the concentrations of solutes in the water. For example, 0.8 kGy given to a 50/50 solution produces > 200 μmol L^−1^ of H_2_O_2_ and decreases the concentration of H_2_ and O_2_ by 38 % and 26 %, respectively. The part of the change in the differential exothermicity with increased dose which is due mainly to the increase in H_2_O_2_, as opposed to the decrease in H_2_ and O_2_, was determined by simulating the radiolysis of a solution saturated with a 50/50 ratio and in which the concentrations of H_2_ and O_2_ were forced to remain constant at the values they had at zero dose. This simulates water in equilibrium with an infinite gas volume of a 50/50 mixture of H_2_ and O_2_. The results are the open circles in Fig. 3, which show that the changes in the exothermicity with accumulated dose in the other curves in Fig. 3 are mostly due to the removal of H_2_ and O_2_. It is noteworthy that, for a first dose of 3 Gy, the exothermicity is quite insensitive to the H_2_/O_2_ ratio. In fact, for the first 3.1 Gy, the exothermicities of all the solutions in Fig. 3 lie within the extremes of the value (2.5 ± 0.07) %, proof of the insensitivity, at low accumulated dose, of a stirred water calorimeter, with a large gas space, to variations in the H_2_/O_2_ ratio.

Simulations carried out for a 43/57 mixture showed that this solution, while not suitable for doses of several kGy, can still be used up to 400 Gy to compare to other solutions such as pure water and H_2_-saturated water. Between 0 Gy and 400 Gy, a high enough dose to allow about a hundred measurements, the heat defect of a 43/57 solution is always in the range 2.5 % to 2.7 %. Under normal operating conditions, the nonuniform dose rate across the vessel and the small gas bubble in the vessel will increase the range only slightly and the heat defect will remain within the range 2.5 % to 2.8 % between 0 Gy and 400 Gy.

### 3.4 Pure Water

At very low doses, the radiolysis of pure water is limited almost entirely to the conversion of water into H_2_O_2_, and H_2_, and is about 5 % endothermic. At higher doses, O_2_ becomes a more significant product but remains less than H_2_ or H_2_O_2_. As the concentrations of H_2_O_2_, H_2_, and O_2_ increase, they participate increasingly in back reactions and at some dose a steady state is reached where, with further irradiation, their removal by back reactions equals their production. At a steady state, the differential heat defect is zero. Fletcher calculated the endothermicity versus accumulated dose for pure water irradiated at several dose rates [19]. Our simulations, using Fletcher’s model, reproduced his results. Fletcher did not report the differential heat defect. He reported the endothermicity for a single dose equal to the total accumulated dose. The differential endothermicity, i.e., the endothermicity expected for a small dose delivered at a particular value of accumulated dose, is a more useful quantity for water calorimetry. A differential heat defect of 0.001 can be considered to be zero for practical water calorimetry. By computer simulation, the doses required to reach a differential heat defect of 0.001, for pure water with no gas space, are a few Gy for both Fletcher’s model and our model. The exact values depend on the dose rate and are shown in Table 4.

In pure water, trace impurities can play an important role in the results because there are no scavengers to remove the reactive species. Previous studies led us to conclude that organic impurities that react with OH radicals are the biggest problem [2, 5]. However, O_2_ is also an obvious impurity and levels as high as 10^−6^ mol L^−1^ are not surprising in the absence of rigorous precautions [20]. Table 4 shows that O_2_, at an initial concentration of 10^−7^ mol L^−1^ in a calorimeter with no gas space, increases the dose required to reach a steady state to 30 Gy or more.

**Fig. 4:**
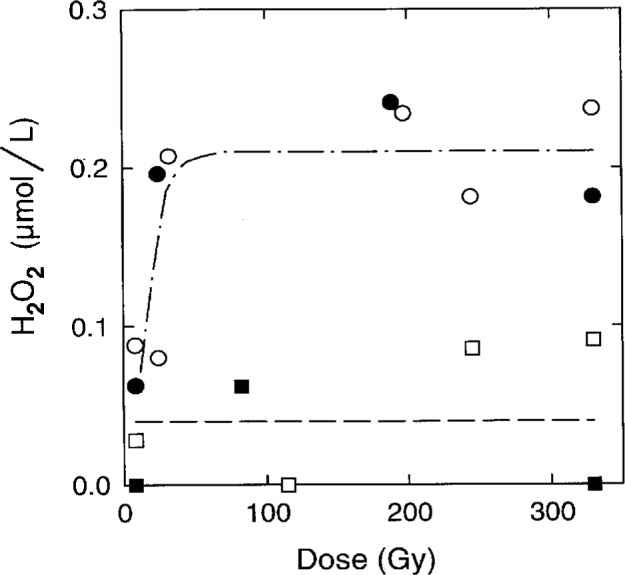
The concentration of H_2_O_2_ for pure and H_2_-saturated water as a function of dose. Pure water at 21 °C is indicated by ○ and at 0 °C is indicated by ●; the dash-dot line is only an aid to the eye. H_2_-saturated water at 21 °C is indicated by □ and at 0 °C is indicated by ■; the dashed line is only an aid to the eye.

We measured the production of H_2_O_2_ at 21 °C and 0 °C in irradiated pure water: 3 ml of water, in a glass tube with an internal diameter of 14 mm, was deaerated by bubbling with ultrapure argon, sealed, and then irradiated at 8 Gy min^−1^. A steady state concentration of 0.2 μmol L^−1^ of H_2_O_2_ was reached by 25 Gy at both 21 °C and 0 °C (Fig. 4). At 8 Gy, the concentration of H_2_O_2_ was well below 0.2 μmol L^−1^ despite the prediction in Table 4 that a steady state should be reached by 8 Gy. Model III and model II predicted a steady state concentration of 0.1 μmol L^−1^ and 0.3 μmol L^−1^ of H_2_O_2_, respectively, for pure water at 21 °C. The results in Fig. 4 show that deaerated water of our present quality in a sealed water calorimeter would require a pre-irradiation of about 25 Gy to arrive at a steady state at both 21 °C and 0 °C. Presumably, the same applies to water calorimetry at 4 °C since the production of H_2_O_2_ versus dose is the same at 21 °C and 0 °C (Fig. 4).

### 3.5 H_2_-Saturated Water

The water calorimetry of H_2_-saturated water in our calorimeter showed an exothermicity of (0.36 ± 0.08) % for ^60^Co radiation, assuming H_2_/O_2_ water to have an exothermicity of 2.4 %. This is surprising for several reasons: (a) H_2_-saturated water is expected to be close to a steady state by the end of the first irradiation period consisting of a 15 Gy dose (Fig. 4); (b) an exothermicity of 0.0 % had been measured for H_2_-saturated water in a 20 MV x-ray beam, also based on an exothermicity of 2.4 % for H_2_/O_2_ water [5]; and (c) organic impurities should be less of a problem in H_2_-saturated water than in pure water because H_2_ scavenges OH radicals. Therefore, we decided to investigate O_2_ contamination of H_2_-saturated water as a possible source of exothermicity. The measurement of O_2_ in water and gas was done using an EIT probe. When 100 mL of water was bubbled with H_2_ at a flow rate of 200 cm^3^ min^−1^, the concentration of O_2_ in the water reached 2 × 10^−8^ mol L^−1^ by 40 min and 1 × 10^−8^ mol L^−1^ by 100 min. However, a small amount of O_2_ (from the air), estimated to be 3 × 10^15^ molecules per minute, was shown to leak into the gas space of the calorimeter after closing the valves. The half-life for equilibration of O_2_ between gas and water is 12 min [5] and, at equilibrium, 94 % of the total O_2_ is in the gas phase. Using these values, simulations predicted that an O_2_ leak of this magnitude would result in an exothermicity of 0.4 % for H_2_-saturated water in a typical water calorimetry measurement. Thus, oxygen entering the vessel at the estimated rate appeared to be a plausible explanation for the discrepancy between experiment and the model prediction of the heat defect. As a test of this hypothesis, changes were made to the gas bubbling tubing in order to eliminate O_2_ leakage from all sources except for the glass-to-metal seal of the vessel and four 4 mm o.d. and one 50 mm o.d. O-rings, and one 5 mm o.d. dynamic O-ring (for the stirrer) which are integral parts of the calorimeter. After these changes, 3 water calorimetry sets with H_2_-saturated water and 2 sets with H_2_/O_2_ water, gave an average exothermicity of (0.32 ± 0.14) % for H_2_-saturated water, essentially the same result as before the change. Therefore, we were not able to confirm that the disagreement between H_2_/O_2_ water and H_2_-saturated water was due to air entering the calorimeter.

The production of H_2_O_2_ was measured in H_2_-saturated water irradiated at 21 °C and 0 °C using the $I_{3}^{-}$ method and the same procedure as described above for pure water. The results are shown in Fig. 4. The steady state concentration of H_2_O_2_ in H_2_-saturated water was about 0.05 μmol L^−1^ at both temperatures and a steady state was reached by 8 Gy, the lowest dose given. Computer simulations predicted a steady state concentration of 0.02 μmol L^−1^. Therefore, for the same quality of H_2_-saturated water in a sealed water calorimeter, a steady state is expected below 8 Gy at both 21 °C and 0 °C and, by interpolation, at 4 °C as well.

**Fig. 5:**
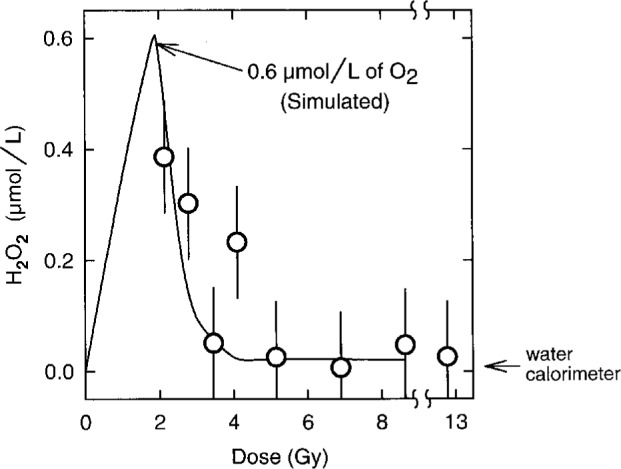
Concentration of H_2_O_2_ versus dose for H_2_-saturated water containing a trace of O_2_ and irradiated in a vessel with no gas space. The data points represent experimental measurements for solutions containing (5 ± 1) × 10^−7^ mol L^−1^ of O_2_. The solid line represents a simulation for 6 × 10^−7^ mol L^−1^ of O_2_. The arrow outside the graph indicates the average measured value for H_2_-saturated water irradiated to doses of about 13 Gy in our water calorimeter under standard operating conditions.

H_2_O_2_ production in irradiated H_2_-saturated water will be enhanced when traces of O_2_ are present, but a sufficient dose will reduce the concentrations of O_2_ and H_2_O_2_ to steady state levels. To demonstrate this, we irradiated H_2_-saturated water which had been prepared with (0.5 ± 0.1) μmol L^−1^ of O_2_ in a vessel with no gas space. The concentration of H_2_O_2_ versus dose was measured. As shown in Fig. 5, a computer simulation of the radiolysis of water containing 0.6 μmol L^−1^ of O_2_ is in good agreement with experiment. Also shown in Fig. 5 is the average of three measurements of the concentration of H_2_O_2_ in H_2_-saturated water irradiated to about 13 Gy in our calorimeter. Figure 5 demonstrates that the absence of detectable H_2_O_2_ is not infallible proof of a zero heat defect during the irradiation. For example, water, which was ostensibly saturated with H_2_ but containing 0.5 μmol L^−1^ of O_2_, would contain negligible H_2_O_2_ after the first 5 Gy but the irradiation would have been exothermic by 5.37 % compared to endothermic by 0.13 % for 5 Gy in the absence of O_2_. Similarly, a small oxygen leak during measurements might not be detected by a measurement of H_2_O_2_.

### 3.6 Air- and O_2_-Saturated Water

In the past, air- and oxygen-saturated water have been used for water calorimetry [6]. The radiation-induced chemical changes in both systems are almost identical. Purified water, in equilibrium with air, would seem to be attractive for water calorimetry because of its simplicity. Instead, it has proven to be a difficult system to deal with, both experimentally and theoretically. Allen and Holroyd [21] investigated the effect of different purification methods on the radiolysis of air-saturated water. They found that water, distilled from acid dichromate and alkaline permanganate as part of a triple-distillation, was suitable for Fricke dosimetry but not for a determination of *G*(H_2_O_2_). The values of *G*(H_2_O_2_) for this water were 57 % and 45 % higher, at 30 Gy and 60 Gy respectively, than measured for water of improved quality. Our measured values of *G*(H_2_O_2_) for O_2_-saturated water were similar to their high values. In order to reduce impurities to a suitably low level, Allen and Holroyd thoroughly cleaned the distillation apparatus, allowed only air which had passed through silica gel and activated charcoal filters to contact the purified water, did not allow plastic tubing to contact the water, steam-cleaned the sample vessels, pre-irradiated them to a brown color, and kept them filled with their purest water when not in use. Also, a crucial part of their procedure was pre-irradiation of the water, followed by destruction of the radiation-produced H_2_O_2_ by photolysis with a low pressure mercury lamp. Even under the cleanest conditions, Allen and Holroyd found an excess of 0.5 μmol L^−1^ of H_2_O_2_ at the lowest doses. This excess suggests that traces of organic impurities in their purest system reacted with OH radicals due to the lack of an OH scavenger in air-saturated water. It is possible that some of the excess peroxide in our measurements and those of Allen and Holroyd could have been organic peroxides rather than H_2_O_2_. Without knowing the source of the excess H_2_O_2_ or organic peroxide, it is not possible to calculate the excess exothermicity.

Using their best water, Allen and Holroyd [21] measured *G*(H_2_O_2_) to be 1.23 in air-saturated water at pH 5. No other reliable value has been reported for air- or O_2_-saturated water near neutral pH at low dose rates. Allen and Holroyd used a ^60^Co source and Fricke dosimetry. However, *εG*(Fe^3+^) is probably 1.7 % smaller [11, 13] than assumed by Allen and Holroyd [21, 22]. This reduces their value for *G*(H_2_O_2_) to 1.21. We assume that *G*(H_2_O_2_) for air- or O_2_-saturated water lies within the range 1.19–1.23.

We carried out computer simulations of the irradiation of air-saturated water, which contains 2.9 × 10^−4^ mol L^−1^ of O_2_ and has a pH of 5, to compare to *G*(H_2_O_2_) = 1.21 (note that the N_2_ dissolved in air-saturated water is considered to be unreactive). Model III predicted *G*(H_2_O_2_) = 0.95 and model II predicted 1.18. In order to ascertain what caused the 24 % difference between the predictions of models III and II, both models were reduced to their most important reactions. Model III was reduced to reactions 5, 11, 15–20, 28, and 35–38 (Table 1). The same reactions, but missing reactions 37 and 38, constituted the reduced model for the version II, which retained its published *G*-values and rate constants [5]. Both reduced models predicted values of *G*(H_2_O_2_) that were 1.02 times larger than predicted by their respective full models. A comparison of these two reduced models showed that the 24 % difference was distributed over several factors. About 51 % of the difference is due to differences in *G*-values, about 31 % is due to reactions 37 and 38 which are missing in model II, and 18 % is due to differences in the rate constants. Fricke [23] showed that, up to about 70 Gy, *G*(H_2_O_2_) is independent of the oxygen concentration in the water between air-saturated water and O_2_-saturated water. Model III bore this out but model II predicted that *G*(H_2_O_2_) drops to 1.07 for O_2_-saturated water at a pH of 7.0. The decrease in going from air-saturated to O_2_ -saturated water for version II is due to the change in pH from 5.0 to 7.0 in contrast to Fricke’s finding that *G*(H_2_O_2_) is independent of pH from 3 to 7.5. We conclude that the use of air- or oxygen-saturated water in water calorimetry is inadvisable because of the lack of a satisfactory understanding of its radiolysis, both experimentally and theoretically.

Water calorimetry using insufficiently pure water has led to excess exothermicity for air- and oxygen-saturated water [2, 5, 7]. We examined this effect by simulating the radiolysis of air-saturated water at pH 5 and a dose rate of 0.1 Gy s^−1^ (the conditions of Allen and Holroyd) in the absence and presence of a model impurity, formic acid. Twelve reactions [17, 24] were added to the model to account for the presence of formic acid but only 2 reactions,

$$\begin{array}{c} \mathrm{OH}+\mathrm{HCOOH}\to \mathrm{COOH}+H_{2}O\\ k=1.3\times 10^{8}L\;{\mathrm{mol}}^{-1}\;s^{-1} \end{array}$$ (8)

and

$$\begin{array}{c} \mathrm{COOH}+O_{2}\to {\mathrm{HO}}_{2}+{\mathrm{CO}}_{2}\\ k=2.4\times 10^{9}L\;{\mathrm{mol}}^{-1}\;s^{-1}, \end{array}$$ (9)

were important, and they increased the importance of reactions 6 and 7. Simulations for 5 × 10^−6^ mol L^−1^ of formic acid roughly followed our earliest water calorimetry measurements with O_2_-saturated water [2] in which the initial endothermicity was about 0.0 % but changed to a steady value of about 2 % endothermic after about 100 Gy. It should be noted that 5 × 10^−6^ mol L^−1^ of formic acid is about 20 times the total organic carbon measured in the water which emerges from our Milli-Q UV unit. These simulations support the hypothesis that organic impurities can make the calorimetry of air-saturated water more exothermic, but the impurities from laboratory to laboratory could be different and are unknown.

## 4. Summary and Conclusions

Water calorimetry has been carried out with pure water and several aqueous solutions. Each solution has advantages and disadvantages which favor different types of calorimeters and procedures, i.e., motionless water versus stirred water, large gas space versus no gas space, and low accumulated dose versus high accumulated dose. Now that these factors have been investigated by experiment, chemical analysis, and by computer simulation, a number of important conclusions have emerged.

1. Water quality is a major concern in water calorimetry. Modern water purification systems can produce very high quality water in plentiful amounts without distillation. However, subsequent exposure of this water to air and other materials easily introduces impurities in sufficient amounts to affect the heat defect. Extreme cleanliness is recommended. The addition of H_2_ to compete with the impurities for reactive species can be advantageous.
2. Simulations indicated that, ideally, less than 10 Gy should bring initially pure water to a steady state but, in our case, measurements of H_2_O_2_ production showed that a steady state was reached somewhere between 8 Gy and 25 Gy. The H_2_O_2_ production was similar at 21 °C and 0 °C and, by interpolation, the same should hold for pure water at 4 °C. Simulations showed that, if pure water is contaminated with O_2_ at 10^−7^ mol L^−1^ or greater, there is a significant increase in the dose required to bring about a steady state.
3. The production of H_2_O_2_ by radiolysis of H_2_/O_2_ water in our stirred water calorimeter as calculated by computer simulation and measured by chemical analysis were in excellent agreement. This agreement and a comparison of the water calorimetry of a number of aqueous solutions justifies the value of (2.4 ± 0.5) % assigned to the heat defect of H_2_/O_2_ water in our calorimeter.
4. The irreprodicible results obtained by Domen [1] using H_2_/O_2_ water in a sealed water calorimeter have been studied in detail. Preliminary work, which is summarized in Domen’s paper, suggested that the discrepancies were caused by large variations in the ratio of H_2_/O_2_ in the gas mixtures, which were intended to have been 50/50 mixtures. The more detailed simulations reported here confirm this result and show that water saturated with a 50/50 mixture, which has been successfully used in a stirred water calorimeter with a large gas space, is not suitable for large accumulated doses in the absence of a gas space. However, a 43/57 mixture could be useful in the absence of a gas space since an almost constant heat defect is predicted for the first 400 Gy.
5. H_2_ scavenges OH radicals. This gives H_2_-saturated water an advantage over pure water. Measurements of H_2_O_2_ at 21 °C and 0 °C confirmed that H_2_ -saturated water reaches a steady state below 8 Gy. However, traces of O_2_ must be guarded against because they cause an exothermic response.
6. It was shown experimentally, by simulation, and by reference to the work of Allen and Holroyd [21], that water calorimetry with air- or O_2_-saturated water is extremely sensitive to the effects of impurities. Consequently, the use of air- or O_2_-saturated water is not recommended for any type of water calorimeter.

## Figures and Tables

**Table 1:** Model III: reactions and rate constants (25 °C) [15]

	Reactions^a^			Rate constants^b^
1.	$e_{\mathrm{aq}}^{-}+e_{\mathrm{aq}}^{-}$	→	H_2_ + OH^−^ OH^−^	6.44 × 10^9^
2.	$e_{\mathrm{aq}}^{-}+H$	→	H_2_ + OH^−^	2.64 × 10^10^
3.	$e_{\mathrm{aq}}^{-}+\mathrm{OH}$	→	OH^−^	3.02 × 10^10^
4.	$e_{\mathrm{aq}}^{-}+H_{2}O_{2}$	→	OH^−^ + OH	1.41 × 10^10^
5.	$e_{\mathrm{aq}}^{-}+O_{2}$	→	$O_{2}^{-}$	1.79 × 10^10^
6.	$e_{\mathrm{aq}}^{-}+O_{2}^{-}$	→	${\mathrm{HO}}_{2}^{-}+{\mathrm{OH}}^{-}$	1.30 × 10^10^
7.	$e_{\mathrm{aq}}^{-}+{\mathrm{HO}}_{2}$	→	${\mathrm{HO}}_{2}^{-}$	1.28 × 10^10^
8.	H + H	→	H_2_	5.43 × 10^9^
9.	H + OH	→	H_2_O	1..53 × 10^10^
10.	H + H_2_O_2_	→	OH + H_2_O	5.16 × 10^7^
11.	H + O_2_	→	HO_2_	1.32 × 10^10^
12.	H + HO_2_	→	H_2_O_2_	9.98 × 10^9^
13.	$H+O_{2}^{-}$	→	${\mathrm{HO}}_{2}^{-}$	9.98 × 10^9^
14.	OH + OH	→	H_2_O_2_	4.74 × 10^9^
15.	OH + H_2_	→	H + H_2_O	4.15 × 10^7^
16.	OH + H_2_O_2_	→	H_2_O + HO_2_	2.87 × 10^7^
17.	OH + HO_2_	→	H_2_O + O_2_	1.08 × 10^10^
18.	$H+O_{2}^{-}$	→	OH^−^ + O_2_	1.10 × 10^10^
19.	HO_2_ + HO_2_	→	H_2_O_2_ + O_2_	6.64 × 10^5^
20.	${\mathrm{HO}}_{2}+O_{2}^{-}$	→	H_2_O_2_ + O_2_ + OH^−^	7.58 × 10^7^
21.	H_2_O	→	H^+^ + OH^−^	1.95 × 10^−5^
22.	$H^{+}+{\mathrm{OH}}^{-}$	→	H_2_O	1.10 × 10^11^
23.	H_2_O_2_	→	$H^{+}+{\mathrm{HO}}_{2}^{-}$	7.86 × 10^−2^
24.	$H^{+}+{\mathrm{HO}}_{2}^{-}$	→	H_2_O_2_	4.78 × 10^10^
25.	$H_{2}O_{2}+{\mathrm{OH}}^{-}$	→	${\mathrm{HO}}_{2}^{-}+H_{2}O$	1.27 × 10^10^
26.	${\mathrm{HO}}_{2}^{-}+H_{2}O$	→	H_2_O_2_ + OH^−^	1.36 × 10^6^
27.	H	→	$e_{\mathrm{aq}}^{-}+H^{+}$	6.32 × 10^0^
28.	$e_{\mathrm{aq}}^{-}+H^{+}$	→	H	2.25 × 10^10^
29.	$e_{\mathrm{aq}}^{-}+H_{2}$	→	H + OH^−^	1.55 × 10^1^
30.	$H+{\mathrm{OH}}^{-}$	→	$e_{\mathrm{aq}}^{-}+H_{2}O$	2.49 × 10^7^
31.	OH	→	H^+^ + O^−^	7.86 × 10^−2^
32.	$H^{+}+O^{-}$	→	OH	4.78 × 10^10^
33.	$H+{\mathrm{OH}}^{-}$	→	O^−^ + H_2_O	1.27 × 10^10^
34.	$O^{-}+H_{2}O$	→	OH^−^ + OH	1.36 × 10^6^
35.	HO_2_	→	$O_{2}^{-}+H_{2}$	7.14 × 10^5^
36.	$O_{2}^{-}+H^{+}$	→	HO_2_	4.78 × 10^10^
37.	${\mathrm{HO}}_{2}+{\mathrm{OH}}^{-}$	→	$O_{2}^{-}+H_{2}O$	1.27 × 10^10^
38.	$O_{2}^{-}+H_{2}O$	→	HO_2_ + OH^−^	1.36 × 10^6^
39.	$O^{-}+H_{2}$	→	H + OH^−^	1.21 × 10^8^
40.	O^−^ + H_2_O_2_	→	$O_{2}^{-}+H_{2}O$	5.53 × 10^8^
41.	$\mathrm{OH}+{\mathrm{HO}}_{2}^{-}$	→	OH^−^ + HO_2_	8.29 × 10^9^
42.	OH + O^−^	→	${\mathrm{HO}}_{2}^{-}$	7.60 × 10^9^
43.	$e_{\mathrm{aq}}^{-}+{\mathrm{HO}}_{2}^{-}$	→	O^−^ + OH^−^	3.50 × 10^9^
44.	$e_{\mathrm{aq}}^{-}+O^{-}$	→	OH^−^ + OH^−^	2.31 × 10^10^
45.	O^−^ + O_2_	→	$O_{3}^{-}$	3.70 × 10^9^
46.	$O_{3}^{-}$	→	O_2_ + O^−^	2.68 × 10^3^
47.	$O+{\mathrm{HO}}_{2}^{-}$	→	$O_{2}^{-}+{\mathrm{OH}}^{-}$	4.00 × 10^8^
48.	$O-O_{2}^{-}$	→	OH^−^ + OH^−^ + O_2_	6.00 × 10^8^
49.	HO_2_ + H_2_O_2_	→	OH + H_2_O + O_2_	5.00 × 10^−1^
50.	$O_{2}^{-}+H_{2}O_{2}$	→	OH^−^ + OH + O_2_	1.30 × 10^−1^

a All reactions are second order except for reactions 21, 23, 27, 31, 35, and 46, which are first order.

b Second order rate constants are in the unit L mol^−1^ s^−1^. First order rate constants are in the unit s^−1^.

**Table 2:** Model III: *G*-values of species [15]

Species	*G*-value^a^ at 21 C [(100 eV)^−1^]	*G*-value^a^ at 25 °C [(100 eV) ^−1^]
H_2_	0.444	0.447
H_2_O_2_	0.648	0.646
$e_{\mathrm{aq}}^{-}$	2.630	2.645
H	0.568	0.572
OH	2.790	2.819
OH^−^	0.430	0.430
H^+^	3.060	3.075
H_2_O	−4.516	−4.541

a The number of significant figures is more than is warranted by the original determinations but is needed for computer simulations in order that the number of H atoms and the number of O atoms in the solution remain constant throughout a simulation.

**Table 3:** Measurement of H_2_O_2_ in H_2_/O_2_ water

Dose (Gy)	H_2_O_2_, calculated (μmol L^−1^)	H_2_O_2_, measured (μmol L^−1^)	Δ^a^ (%)
40.68	14.32	14.02	2.1
40.67	14.31	14.08	1.7
81.27	28.17	27.59	2.1

a Fractional difference between the measured and calculated concentrations of H_2_O_2_.

**Table 4:** Dose to reach 0.1 % endothermicity in pure water, with and without traces of O_2_

O_2_	model	Dose at 1 Gy min^−1^ (Gy)	Dose at 20 Gy min^−1^ (Gy)
10^−7^ mol L^−1^	III	30	55
10^−8^ mol L^−1^	III	3	9
0 mol L^−1^	III	2	8
0 mol L^−1^	Fletcher	4	8

## Footnotes

1 Certain commercial equipment, instruments, or materials are identified in this paper to foster understanding. Such identification does not imply recommendation or endorsement by the National Institute of Standards and Technology, nor does it imply that the materials or equipment identified are necessarily the best available for the purpose.

2 Throughout this document, “uncertainty” refers to an uncertainty of 1 standard deviation.

3 *ε*(X) is the molar extinction coefficient of the species X in the unit L mol^−1^ cm^−1^ at a specified wavelength. *G*(X) is defined as the number of X (free radicals, atoms, molecules, ions etc.) produced, or destroyed, per 100 eV of absorbed energy [12].
